# Joint QTL analysis of three connected F_2_-crosses in pigs

**DOI:** 10.1186/1297-9686-42-40

**Published:** 2010-11-01

**Authors:** Christine Rückert, Jörn Bennewitz

**Affiliations:** 1 Institute of Animal Husbandry and Breeding, University of Hohenheim, D-70599 Stuttgart, Germany

## Abstract

**Background:**

Numerous QTL mapping resource populations are available in livestock species. Usually they are analysed separately, although the same founder breeds are often used. The aim of the present study was to show the strength of analysing F_2_-crosses jointly in pig breeding when the founder breeds of several F_2_-crosses are the same.

**Methods:**

Three porcine F_2_-crosses were generated from three founder breeds (i.e. Meishan, Pietrain and wild boar). The crosses were analysed jointly, using a flexible genetic model that estimated an additive QTL effect for each founder breed allele and a dominant QTL effect for each combination of alleles derived from different founder breeds. The following traits were analysed: daily gain, back fat and carcass weight. Substantial phenotypic variation was observed within and between crosses. Multiple QTL, multiple QTL alleles and imprinting effects were considered. The results were compared to those obtained when each cross was analysed separately.

**Results:**

For daily gain, back fat and carcass weight, 13, 15 and 16 QTL were found, respectively. For back fat, daily gain and carcass weight, respectively three, four, and five loci showed significant imprinting effects. The number of QTL mapped was much higher than when each design was analysed individually. Additionally, the test statistic plot along the chromosomes was much sharper leading to smaller QTL confidence intervals. In many cases, three QTL alleles were observed.

**Conclusions:**

The present study showed the strength of analysing three connected F_2_-crosses jointly. In this experiment, statistical power was high because of the reduced number of estimated parameters and the large number of individuals. The applied model was flexible and was computationally fast.

## Background

Over the last decades, many informative resource populations in livestock breeding have been established to map quantitative trait loci (QTL). Using these populations, numerous QTL for many traits have been mapped [1]. However, the mapping resolution of these studies is usually limited by the size of the population. One way to increase the number of individuals is to conduct a joint analysis of several experimental designs. In dairy cattle breeding, a joint analysis of two half-sib designs with some overlapping families has been performed by Bennewitz et al. [2] and has shown that a combined analysis increases statistical power substantially, due to the enlarged design and especially due to increased half-sib family size. In pig breeding, a joint analysis has been successfully implemented by Walling et al. [3] in which seven independent F_2_-crosses have been analysed in a combined approach for one chromosome. The mapping procedure developed by Haley et al. [4] was used where some breeds are initially grouped together in order to fulfil the assumption of the line cross approach (i.e. two founder lines are fixed for alternative QTL alleles). Further examples can be found in Kim et al. [5] and Pérez-Enciso et al. [6], both using pig crosses, or in Li et al. [7] using laboratory mouse populations.

Analysing several F_2_-crosses jointly could be especially useful when the founder breeds used for the crosses are the same in all the designs. This situation can occur in plant breeding, where crosses are produced from a diallel design of multiple inbred lines (e.g. Jansen et al. [8]). Although rare in livestock breeding, one example is the experiment described by Geldermann [9]. For this kind of experiment Liu and Zeng [10] have proposed a flexible multiallelic mixture model, which estimates an additive QTL effect for each founder line and a dominant QTL effect for each founder line combination. They have estimated their model by adopting maximum likelihood using an EM algorithm.

The aim of the present study was to conduct a joint genome scan covering the autosomes for three porcine F_2_-crosses derived from three founder breeds. For this purpose, the method of Liu and Zeng [10] was modified in order to include imprinting effects. The effect of a combined analysis was demonstrated by comparing the results for three traits with those obtained when the three crosses were analysed separately.

## Methods

### Connected F_2_-crosses

The experimental design is described in detail by Geldermann et al. [9] and only briefly reminded here. The first cross (MxP) was obtained by mating one Meishan (M) boar with eight Pietrain (P) sows. The second cross (WxP) was generated by mating one European wild boar (W) with nine P sows, some of which were the same as in the MxP cross. The third cross (WxM) was obtained by mating the same W boar with four Meishan (M) sows. The number of F_1_-individuals in the MxP, WxP and WxM crosses was 22, 28 and 23, respectively and the the number of F_2_-individuals was 316, 315 and 335, respectively. The number of sires in the F_1_-generation was between two and three. The joint design was built by combining all three designs. All individuals were kept on one farm; housing and feeding conditions have been described by Müller et al. [11]. All F_2_-individuals were phenotyped for 46 traits including growth, fattening, fat deposition, muscling, meat quality, stress resistance and body conformation, see [11] for further details. In this study, we investigated three traits i.e. back fat depth, measured between the 13^th ^and 14^th ^ribs, daily gain and carcass weight. The phenotypes were pre-corrected for the effect of sex, litter, season and different age at slaughtering before QTL analysis. The means and standard deviations of the observations are given in Table 1. There is substantial variation within and between crosses for all three traits. Altogether 242 genetic markers (mostly microsatellites) were genotyped, covering all the autosomes, with a large number of overlapping markers in the crosses. Both sex chromosomes were excluded from the analysis because they deserve special attention (Pérez-Enciso et al. [6]).

**Table 1:** Number of observations (n), mean, standard deviation (Sd), minimum (Min) and maximum (Max) of the phenotypic observations and coefficient of variation (CV)

Trait	Cross	n	Mean	Sd	Min	Max	CV
Back fat depth [mm]	MxP	316	21.96	6.94	6.7	43.3	31.59
	WxP	315	16.76	5.85	5.3	37.3	34.92
	WxM	335	31.62	8.62	6.0	54.7	27.25
	Joint	966	23.61	9.54	5.3	54.7	40.40

Daily gain [g]	MxP	316	589.49	132.03	174.0	951.0	22.40
	WxP	315	528.78	107.83	125.0	790.0	20.39
	WxM	335	456.65	94.14	143.0	741.0	20.61
	Joint	966	523.63	124.61	125.0	951.0	23.80

Carcass weight [kg]	MxP	316	76.22	14.19	42.2	109.6	18.62
	WxP	315	57.14	12.60	19.7	89.2	22.05
	WxM	335	54.75	11.71	20.8	86.8	21.38
	Joint	966	62.55	16.02	19.7	109.6	25.61

### Linkage maps and information content

A common linkage map was estimated using Crimap [12]. Due to the large number of overlapping markers these calculations were straightforward. It was assumed that two founder breeds (breed *i *and *j*, with *i *and *j *being breed M, P, or W) of a single cross are divergent homozygous at a QTL, i.e. showing only genotype *Q_i_Q_i _*and *Q_j_Q_j_*, respectively. Although the three breeds in this study are outbred breeds, this assumption holds approximately, because the breeds have a very different history and are genetically divergent (see also Haley et al. [4]). Subsequently, for each F_2_-individual of a certain cross four genotype probabilities $pr(Q_{i}^{p}Q_{i}^{m})$, $pr(Q_{j}^{p}Q_{i}^{m})$, $pr(Q_{i}^{p}Q_{j}^{m})$ and $pr(Q_{j}^{p}Q_{j}^{m})$ were calculated for each chromosomal position. The upper subscript denotes the parental origin of the alleles (i.e. paternal (*p*) or maternal (*m*) derived) and the lower subscript denotes the breed origin of the alleles (i.e. breed *i *or *j*). These probabilities were estimated using a modified version of Bigmap [13]. This program follows the approach of Haley et al. [4] and uses information of multiple linked markers, which may or may not be fixed for alternative alleles in the breeds. The information content for additive and imprinting QTL effects were estimated for each chromosomal position, using an entropy-based information measure as described by Mantey et al. [14]. The information content for the additive QTL effect represents the probability that two alternative QTL homozygous genotypes can be distinguished, given the individuals are homozygous. Similarly, the imprinting information content denotes the probability that two alternative heterozygous QTL genotypes can be separated, given that the individuals are heterozygous. The information content was solely used to assess the amount of information available to detect QTL and was not used for the QTL mapping procedure.

### Genetic and statistical model

On the whole, the genetic model followed the multiallelic model of Liu and Zeng [10], but was extended to account for imprinting. It is assumed that the breeds are inbred at the QTL. The genetic mean was defined as the mean of the *L *= 3 founder breeds. Considering one locus, the mean is

$$\mu =\frac{\sum_{i=1}^{L}g_{ii}}{L},$$

with *g_ii _*being the homozygote genotypic value in breed *i *(*i *= M, P, and W, respectively). Now let us consider haploid populations. The mean of the breeds consisting of paternal derived and maternal derived alleles at the locus is

$$\mu^{p}=\frac{\sum_{i=1}^{L}g_{i}^{p}}{L}\;\text{and}\;\mu^{m}=\frac{\sum_{i=1}^{L}g_{i}^{m}}{L},$$

respectively. The term $g_{i}^{p}$ ($g_{i}^{m}$) denotes the genotypic value of the paternal (maternal) derived allele. The additive effect of the paternal derived and maternal derived allele is $a_{i}^{p}=g_{i}^{p}-\mu^{p}$ and $a_{i}^{m}=g_{i}^{m}-\mu^{m}$, respectively. This imposes the restrictions

(1) $$\sum_{i=1}^{L}a_{i}^{p}=0\;\text{and}\;\sum_{i=1}^{L}a_{i}^{m}=0.$$

In this haploid model, putative imprinting effects will result in different haploid means. However, in a diallelic model the two haploid means are not observable, but become part of the mean as *μ *= *μ*^*p *^+ *μ*^*m*^. Thus the genetic model of the diploid F_2_-population generated from the breeds *i *and *j *is as follows:

(2) $$\left[\begin{array}{c} g_{ii}^{pm}\\ g_{ij}^{pm}\\ g_{ji}^{pm}\\ g_{jj}^{pm} \end{array}\right]=\left[\begin{array}{ccccc} 1 & 1 & 0 & 0 & 0\\ 1 & 0 & 0 & 1 & 1\\ 0 & 1 & 1 & 0 & 1\\ 0 & 0 & 1 & 1 & 0 \end{array}\right]\left[\begin{array}{c} a_{i}^{p}\\ a_{i}^{m}\\ a_{j}^{p}\\ a_{j}^{m}\\ d_{ij} \end{array}\right]+\left[\begin{array}{c} \mu \\ \mu \\ \mu \\ \mu \end{array}\right],$$

where again the upper subscripts denote the parental origin and the lower subscripts denote the breed origin of the alleles. Putative imprinting effects will result in $a_{i}^{p}\neq a_{i}^{m}$. This genetic model was used to set up the statistical model. We used the notation of Liu and Zeng [10] for comparison purposes.

(3) $$\begin{array}{c} y_{ijk}=cross_{ij}+(z_{ijk,i}^{p}w_{ijk,i}^{p}+z_{ijk,i}^{m}w_{ijk,i}^{m}+z_{ijk,j}^{p}w_{ijk,j}^{p}+z_{ijk,j}^{m}w_{ijk,j}^{m})a\\ +z_{ijk}^{pm}w_{ijk}^{pm}d+e_{ijk} \end{array}$$

where *y_ijk _*is the phenotypic observation of the *k*th individual in the F_2_-cross derived from breed *i *and *j*. The term *cross_ij _*denotes the fixed effect of the F_2_-cross. It was included in the model (and not in the model for the pre-correction of the data for other systematic effects as described above), because it contains a part of the genetic model (i.e. the mean). The term *e_ijk _*is a random residual with heterogeneous variance, i.e. $e_{ijk}\sim N(0,\sigma_{ij}^{2})$. Vector **a **contains the additive effects ($a_{1}^{p},a_{1}^{m},...a_{L}^{p},a_{L}^{m}$) and vector **d **contains the dominance effects (*d*_1,2_, *d*_1,3_, ..., *d*_(*L*-1),*L*_). The four **w **terms are row vectors of length 2*L with one element equal to one and the other elements equal to zero. Each **w **term indicates one of the four possible additive effects in **a **that could be observed in the F_2_-individual based on pedigree data. For example, $w_{ijk,i}^{p}$ denotes the putative allele in offspring *ijk *(indicated by first lower subscript *ijk*) inherited paternally (indicated by upper subscript *p*) from line *i *(indicated by second lower subscript *i*). The four *z *terms are scalars and are either zero or one. They indicate if the offspring inherited the corresponding allele from the corresponding parent. For each offspring these four terms sum up to two. Similarly, $w_{ijk}^{pm}$ is a row vector of length *L*, indicating which dominance effect could be possible in the offspring based on pedigree data. The scalar $z_{ijk}^{pm}$ is one if the offspring is heterozygous at the QTL and zero otherwise. The true *z *terms were unknown and therefore calculated from the four estimated QTL-genotype probabilities at each chromosomal position. For example, the term $z_{ijk,i}^{p}$ was set equal to ${\hat{a}}_{M}^{p}={\hat{a}}_{i}^{p}$. The dominance term ($z_{ijk}^{pm}$) was the sum of the two heterozygous genotype probabilities. The statistical model was a multiple linear regression. The residual variance was assumed to be heterogeneous.

In order to avoid an over-parameterisation due to the restrictions shown in (1), the genetic model (2) was re-parameterised taking the restrictions in (1) into account, as shown in Appendix. The final regression was also re-parameterised taking these restrictions into account. Hence, in fact only 2*L-2 = 4 additive effects were estimated (i.e. ${\hat{a}}_{i}^{p},{\hat{a}}_{i}^{m},{\hat{a}}_{j}^{p},{\hat{a}}_{j}^{m}$). The estimated paternal additive effects of the breeds were ${\hat{a}}_{M}^{p}={\hat{a}}_{i}^{p}$, ${\hat{a}}_{P}^{p}={\hat{a}}_{j}^{p}$ and ${\hat{a}}_{W}^{p}=-({\hat{a}}_{i}^{p}+{\hat{a}}_{j}^{p})$, respectively, where the lower subscripts M, P and W denote the three breeds. The same holds true for the maternal additive effects. The combined mendelian additive QTL effects for the three breeds were calculated as ${\hat{a}}_{M}={\hat{a}}_{i}^{p}+{\hat{a}}_{i}^{m}$, ${\hat{a}}_{P}={\hat{a}}_{j}^{p}+{\hat{a}}_{j}^{m}$, and ${\hat{a}}_{W}=-({\hat{a}}_{i}^{p}+{\hat{a}}_{i}^{m}+{\hat{a}}_{j}^{p}+{\hat{a}}_{j}^{m})$.

The model was fitted every cM on the autosomes by adapting the *z *terms accordingly. The test statistic was an *F*-test; the *F*-values were converted into LOD-scores as *LOD *≈ (np*F)/(2*log(10)) with *np *being the number of estimated QTL effects [14], i.e. *np *= 7 (four additive and three dominance effects).

When imprinting is not accounted for, the models (2) and (3) reduce to the proposed model of Liu and Zeng [10]. In this case, *L *- 1 = 2 additive effects are estimated. In this study, this was also solved by using multiple linear regressions with heterogeneous residual variances.

### Hypothesis testing

The highest test-statistic was recorded within a chromosome-segment (for the definition of a chromosome-segment see the next section). The global null hypothesis was that at the chromosomal position with the highest test statistic, every estimated parameter in **a **and **d **is equal to zero. The corresponding alternative hypothesis was that at least one parameter was different from zero. The 5% threshold of the test statistic corrected for multiple testing within the chromosome-segment was obtained using the quick method of Piepho [15]. Once the global null hypothesis was rejected, the following sub-hypotheses were tested at significant chromosomal positions by building linear contrasts.

Test for an additive QTL:

$${\text{H}}_{0}:a_{i}^{p}+a_{i}^{m}=0\;\text{and}\;a_{j}^{p}+a_{j}^{m}=0,{\text{H}}_{1}:a_{i}^{p}+a_{i}^{m}\neq 0\;\text{and}/\text{or}a_{j}^{p}+a_{j}^{m}\neq 0.$$

The test statistic was an *F*-test with two degrees of freedom in the numerator.

Test for dominance at the QTL:

$${\text{H}}_{\text{0}}:d_{ij}=0,{\text{H}}_{1}:d_{ij}\neq 0.$$

The test statistic was an *F*-test with three degrees of freedom in the numerator.

Test for imprinting at the QTL:

$${\text{H}}_{0}:a_{i}^{p}=a_{i}^{m}\;\text{and}\;a_{j}^{p}=a_{j}^{m},{\text{H}}_{1}:a_{i}^{p}\neq a_{i}^{m}\;\text{and}/\text{or}\;a_{j}^{p}\neq a_{j}^{m}.$$

The test statistic was an *F*-test with two degrees of freedom in the numerator. The mode of imprinting (either paternal or maternal imprinting) at the QTL with significant imprinting effects was assessed by comparing the paternal and maternal effect estimates.

The test of the three sub-hypotheses resulted in the three error probabilities *p_add_*, *p_dom_*, and *p_imp _*for additive, dominance and imprinting QTL, respectively. Note that if the global null hypothesis was rejected, at least one of the three sub-null-hypotheses had to be rejected as well. Therefore, correction for multiple testing was done only for the global null hypothesis, and for the sub-null-hypothesis, the comparison-wise error probabilities were reported.

Finally, the number of QTL alleles that could be distinguished based on their additive effects was assessed. This was done by testing the segregation of the QTL in each of the three crosses, considering only additive mendelian effects (i.e. ignoring imprinting and dominance). The corresponding test was:

$${\text{H}}_{0}:a_{i}^{p}+a_{i}^{m}=a_{j}^{p}+a_{j}^{m},{\text{H}}_{1}:a_{i}^{p}+a_{i}^{m}\neq a_{j}^{p}+a_{j}^{m}.$$

Once again an *F*-test was used and was applied for each of the three crosses. If the QTL segregated between two (three) crosses the number of QTL alleles was two (three). Note that it was not possible that a QTL segregated solely in one cross.

### Confidence intervals and multiple QTL

For each significant QTL, a confidence interval was calculated using the one LOD-drop method mentioned in Lynch and Walsh [16]. The lower and upper bounds were then obtained by going from the lower and upper endpoints of the one LOD-drop region to the next left and next right marker, respectively. This procedure worked against the anti-conservativeness of the one LOD-drop off method. The anti-conservativeness was shown by Visscher et al. [17].

The procedure to include multiple QTL in the model is recursive and proceeds as follows. Initially, the genome was scanned and the 5% chromosomes-wise thresholds were estimated. Next the QTL with the highest test statistic exceeding the threshold was included as a cofactor in the model and the genome was scanned again, but excluding the positions within the confidence interval of this QTL. This was repeated until no additional significant QTL could be identified. In each round of cofactor selection, the question of whether the test statistic of previously identified QTL remained above their significance threshold levels was assessed; a QTL was excluded from the model if no longer significant. This can happen if some linked or even unlinked QTL co-segregate by chance (e.g. de Koning et al. [18]) and the strategy used here accounts for this co-segregation. The thresholds were calculated for chromosomes without having a QTL as a cofactor in the model considering the whole chromosome (i.e. 5% chromosome-wise thresholds). If, however, a QTL on a chromosome was already included as a cofactor, the thresholds were estimated for the chromosome segment spanned by a chromosomal endpoint and the next bound of the QTL confidence interval (i.e. 5% chromosome-segment-wise). In case more than one QTL was included as a cofactor on a chromosome, a chromosome-segment between two QTL was spanned by the two neighbouring bounds of the confidence intervals and the threshold was calculated for this chromosome segment. By defining chromosome-segments in this way, multiple QTL on one chromosome were considered. The significance thresholds were determined for the regions on the chromosomes that were scanned for QTL.

### Separate analysis of three crosses

In the study of Geldermann et al. [9], the crosses were analysed separately, but without modelling imprinting. Therefore, in order to show the benefit of the joint analysis, the crosses were analysed again separately, but accounting for imprinting. The following standard model was applied:

(4) $$y_{ijk}=\mu +a*p_{a}+d*p_{d}+imp*p_{im}+e_{ijk},$$

where *μ *is the mean of the F_2_-offpring of the cross, $p_{a}=pr(Q_{i}^{p}Q_{i}^{m})-pr(Q_{j}^{p}Q_{j}^{m})$, $p_{d}=pr(Q_{i}^{p}Q_{j}^{m})+pr(Q_{j}^{p}Q_{i}^{m})$, and $p_{im}=pr(Q_{i}^{p}Q_{j}^{m})-pr(Q_{j}^{p}Q_{i}^{m})$. The terms *a*, *d*, and *im *are the regression coefficients, representing the additive, dominance, and imprinting effects, respectively. The test statistic was an *F*-test; LOD scores were obtained as described above, but using *np *= 3. Chromosome-segment-wise 5% threshold values were obtained again using the quick method explained earlier. Multiple QTL were considered as described above.

## Results

The marker order of the estimated linkage map (see Additional file 1) is in good agreement with other maps. The average information content for additive and imprinting effects was high (about 0.868 and 0.752, respectively, averaged over all individuals and chromosomal positions). This indicated that informative markers were dense enough to detect imprinting effects (which requires a higher marker density [14]).

The results of the joint design (obtained with model (3)) for the traits back fat depth, daily gain and carcass weight are shown in Tables 2, 3, and 4, respectively, and of the separate analysis of the three crosses (obtained with model (4)) are shown in Table 5. For each reported QTL in the joint design (i.e. showing an error probability smaller than 5% chromosome-segment-wise) the estimated QTL position, the confidence interval, and the comparison-wise error probabilities of the sub-hypothesis are given. A sub-hypothesis was declared as significant if the comparison-wise error probability was below 5%. QTL effects are often heavily overestimated due to significance testing (e.g. Göring et al. [19]). Therefore, we did not report these estimates, except for QTL showing imprinting (Table 6). Instead we reported the order of the breed QTL effects in Tables 2, 3, and 4.

**Table 2:** QTL results from the joint design and back fat

SSC	Position	CI^a^	*F*-value	*p_add_*^b^	*p_dom_*^c^	*p_imp_*^d^	Order of effects^e^
1	90	[59.3; 95.8]	3.11	0.0195	0.0762	0.1062	*â_P _> â_M _> â_W_*
1	144	[126.3; 149.6]	6.81	<0.0001	0.0889	0.2779	*â_P _> â_M _> â_W_*
1	179	[149.6; 209.1]	2.80	0.0101	0.1010	0.5290	*â_M _> â_P _= â_W_*
2	13	[0.0; 39.9]	5.01	0.0058	0.5031	<0.0001	*â_M _> â_P _= â_W_*
2	77	[68.0; 81.0]	5.79	<0.0001	0.1947	0.3441	*â_P _> â_M _> â_W_*
6	100	[96.4; 101.2]	6.46	<0.0001	0.0275	0.0587	*â_M _> â_P _= â_W_*
7	83	[75.5; 100.9]	5.81	<0.0001	0.0593	0.0422	*â_W _> â_M _= â_P_*
11	83	[61.0; 93.3]	2.77	0.0094	0.1511	0.0939	*â_P _> â_M _= â_W_*
12	58	[0.0; 84.1]	3.37	0.2599	0.0006	0.2458	*â_M _= â_P _= â_W_*
13	56	[39.2; 81.2]	2.34	0.3950	0.0134	0.1595	*â_M _= â_P _= â_W_*
14	51	[27.5; 60.7]	3.05	0.0107	0.0332	0.0802	*â_M _= â_P _> â_W_*
17	74	[43.6; 97.9]	2.26	0.0199	0.9068	0.0267	*â_M _> â_P _= â_W_*
18	27	[10.9; 43.6]	4.38	<0.0001	0.0251	0.2384	*â_M _= â_P _> â_W_*

^a ^confidence interval; ^b ^comparison-wise error probability for additive effects; ^c ^comparison-wise error probability for dominant effects; ^d ^comparison-wise error probability for imprinting effects; ^e ^*â_P _*estimated effect of Pietrain breed, *â_M _*estimated effect of Meishan breed, *â_W _*estimated effect of the wild boar breed

**Table 3:** QTL results from the joint design and daily gain

SSC	Position	CI^a^	*F*-value	*p_add_*^b^	*p_dom_*^c^	*p_imp_*^d^	Order of effects^e^
1	58	[25.4; 77.3]	3.27	0.0001	0.1850	0.6335	*â_P _> â_M _> â_W_*
1	134	[126.3; 141.7]	6.15	<0.0001	0.1376	0.1203	*â_P _> â_M _> â_W_*
2	8	[0.0; 39.9]	3.17	0.0058	0.0173	0.8928	*â_P _= â_W _> â_M_*
3	58	[50.8; 74.0]	5.39	0.0006	0.0008	0.0241	*â_P _= â_W _> â_M_*
4	93	[85.6; 98.1]	5.15	<0.0001	0.5892	0.7868	*â_P _> â_M _> â_W_*
5	128	[92.2; 150.4]	2.95	0.4389	0.8924	0.0001	*â_M _= â_P _= â_W_*
6	91	[80.0; 112.0]	2.93	0.0110	0.0647	0.1012	*â_P _> â_M _> â_W_*
6	202	[177.9; 235.5]	2.94	0.0441	0.0161	0.1780	*â_W _> â_M _> â_P_*
7	42	[24.8; 94.4]	2.65	0.0080	0.5892	0.0261	*â_M _= â_P _> â_W_*
8	8	[0.0; 34.0]	4.20	<0.0001	0.5782	0.0363	*â_P _> â_M _> â_W_*
9	90	[80.0; 110.1]	2.86	0.0018	0.5195	0.1961	*â_W _> â_M _= â_P_*
9	194	[187.4; 194.6]	3.29	0.0778	0.0011	0.3357	*â_M _= â_p _= â_W_*
10	53	[30.6; 74.1]	2.98	0.6023	0.0044	0.0509	*â_M _= â_P _= â_W_*
15	67	[52.5; 99.4]	2.99	0.0038	0.0655	0.4120	*â_M _= â_P _> â_W_*
16	87	[69.4; 98.0]	3.14	0.2405	0.0043	0.0676	*â_M _= â_P _= â_W_*

^a ^confidence interval; ^b ^comparison-wise error probability for additive effects; ^c ^comparison-wise error probability for dominant effects; ^d ^comparison-wise error probability for imprinting effects; ^e ^*â_P _*estimated effect of Pietrain breed, *â_M _*estimated effect of Meishan breed, *â_W _*estimated effect of the wild boar breed

**Table 4:** QTL results from the joint design and carcass weight

SSC	Position	CI^a^	*F*-value	*p_add_*^b^	*p_dom_*^c^	*p_imp_*^d^	Order of effects^e^
1	89	[77.3; 104.1]	7.94	<0.0001	0.7482	0.0385	*â_P _> â_M _> â_W_*
2	76	[70.6; 81.0]	5.55	<0.0001	0.0143	0.2408	*â_P _> â_M _> â_W_*
3	0	[0.0; 35.9]	3.34	0.0001	0.1644	0.5312	*â_P _> â_M _> â_W_*
3	58	[50.2; 74.0]	3.01	0.0489	0.0064	0.3611	*â_P _= â_W _> â_M_*
4	73	[62.1; 81.0]	6.00	<0.0001	0.2317	0.6112	*â_P _> â_M _> â_W_*
4	97	[87.6; 107.7]	2.64	0.0016	0.3586	0.1014	*â_P _> â_M _> â_W_*
5	120	[110.0; 150.4]	3.05	0.0216	0.7526	0.0022	*â_W _> â_M _= â_P_*
6	87	[80.0; 94.4]	4.38	0.0006	0.0105	0.0800	*â_P _> â_M _> â_W_*
7	36	[0.0; 50.0]	2.60	0.1441	0.0243	0.0415	*â_M _= â_P _= â_W_*
7	59	[36.3; 73.3]	3.63	0.0003	0.0623	0.4030	*â_M _= â_P _> â_W_*
8	13	[0.0; 34.0]	4.80	<0.0001	0.3863	0.0822	*â_P _> â_M _> â_W_*
8	127	[110.1; 151.8]	2.99	0.0191	0.0088	0.6977	*â_P _= â_W _> â_M_*
10	59	[30.6; 74.1]	2.69	0.9783	0.0346	0.0085	*â_M _= â_P _= â_W_*
12	86	[64.5; 109.8]	2.53	0.0070	0.2919	0.0902	*â_P _> â_M _> â_W_*
14	93	[60.7; 105.1]	2.98	<0.0001	0.9244	0.8026	*â_P _> â_M _> â_W_*
16	0	[0.0; 21.2]	3.62	0.4887	0.0438	0.0010	*â_M _= â_P _= â_W_*

^a ^confidence interval; ^b ^comparison-wise error probability for additive effects; ^c ^comparison-wise error probability for dominant effects; ^d ^comparison-wise error probability for imprinting effects; ^e ^*â_P _*estimated effect of Pietrain breed, *â_M _*estimated effect of Meishan breed, *â_W _*estimated effect of the wild boar breed

**Table 5:** QTL results from the three single crosses (MxP, WxP, WxM) for the three traits

Cross	Trait	SSC	Position	CI
MxP	Back fat depth	2	52	[0.0; 78.3]
		6	97	[80.0; 98.3]
		6	100	[98.3; 101.2]
		6	104	[101.2; 124.9]
		12	4	[0.0; 51.0]
WxP		1	135	[126.3; 149.6]
		7	47	[0.0; 73.3]
WxM		1	144	[126.3; 149.6]
		2	78	[52.9; 81.0]

MxP	Daily gain	3	58	[50.8; 74.0]
WxP		1	60	[43.5; 77.3]
		1	90	[77.3; 119.2]
		1	133	[119.2; 141.7]
		2	67	[52.9; 96.0]
		8	0	[0.0; 18.0]
		9	194	[187.4; 194.6]
WxM		7	58	[36.3; 73.3]
		15	66	[52.5; 99.4]

MxP	Carcass weight	2	76	[70.6; 78.3]
		4	82	[27.7; 98.1]
		8	21	[0.0; 49.4]
WxP		1	62	[43.5; 77.3]
		1	133	[110.3; 141.7]
		2	68	[52.9; 81.0]
		2	90	[81.0; 115.1]
		16	0	[0.0; 21.2]
WxM		1	83	[43.5; 95.8]
		1	144	[126.3; 149.6]
		7	63	[50.0; 75.2]

**Table 6:** Additive QTL effects and mode of imprinting for QTL showing significant imprinting effects: results from the joint design

Trait	SSC	Pos.	${â}_{M}^{p}{}^{*}$		${â}_{M}^{m}$		${â}_{P}^{p}$		${â}_{P}^{m}$		${â}_{W}^{p}$		${â}_{W}^{m}$		Mode
Back fat depth	2	13	**1.30**	(0.65)	0.10	(0.65)	-1.18	(1.00)	0.75	(1.03)	-0.12	(1.61)	-0.85	(1.65)	nc
	7	83	**-1.28**	(0.64)	**-3.30**	(0.67)	-0.002	(0.99)	**-2.97**	(1.05)	1.28	(1.59)	**5.26**	(1.67)	pat
	17	74	**2.42**	(0.67)	-0.41	(0.70)	**3.31**	(1.11)	-1.33	(1.19)	**-5.72**	(1.74)	1.73	(1.85)	mat

Daily gain	3	58	**-24.99**	(9.52)	10.69	(9.20)	-4.67	(18.27)	**35.03**	(16.05)	29.66	(26.62)	-45.72	(24.19)	nc
	5	128	**-30.74**	(9.77)	15.29	(10.17)	-28.06	(16.38)	-2.62	(16.92)	**58.80**	(25.07)	-12.67	(25.92)	mat
	7	42	3.98	(9.42)	**34.75**	(10.14)	19.17	(15.65)	26.04	(16.81)	-23.15	(23.61)	**-60.79**	(25.47)	pat
	8	8	16.73	(10.51)	-7.26	(10.82)	**71.24**	(17.96)	3.81	(18.63)	**-87.97**	(27.2)	3.45	(28.01)	mat

Carcass weight	1	89	**6.08**	(1.36)	**3.22**	(1.30)	**10.41**	(2.33)	**10.12**	(2.23)	**-16.49**	(3.55)	**-13.33**	(3.40)	mat
	5	120	**-3.76**	(0.97)	0.01	(0.99)	**-4.36**	(1.66)	-2.10	(1.69)	**8.12**	(2.53)	2.09	(2.57)	mat
	7	36	1.07	(1.52)	2.31	(1.51)	**5.79**	(2.75)	1.22	(2.66)	-6.86	(4.04)	-3.54	(4.01)	nc
	10	59	**2.47**	(1.09)	-2.20	(1.21)	**4.59**	(1.90)	-4.01	(2.07)	**-7.06**	(2.87)	6.21	(3.17)	mat
	16	0	**2.90**	(1.05)	-1.70	(1.10)	**6.31**	(1.78)	-3.42	(1.84)	**-9.21**	(2.72)	5.11	(2.82)	mat

Significant additive effects are written in bold face; standard errors are given in parenthesis;

*upper subscript denotes parental origin (paternal or maternal derived) and lower subscript denotes breed (M, P or W); mat = maternal, pat = paternal, nc = not consistent

Thirteen QTL were found for back fat depth (see Table 2) of which 11 showed a significant additive effect, five significant dominant effects and three a significant imprinting effect. The QTL on SSC12 and SSC13 were only significant because of their dominance effects. For three QTL, three alleles could be identified based on their combined additive effect. In all three cases the effect of the P breed allele was highest, followed by the effect of the M breed allele. For other QTL, the effect of the M breed allele was higher compared to that of the P and W breeds, whereby P and W were often the same when only two QTL alleles could be separated. Naturally, for those QTL without a significant additive effect no order of breed allele effects could be observed. For daily gain, 15 QTL were mapped of which 11 showed a significant additive, six a significant dominant and four a significant imprinting effect (Table 3). The QTL on SSC5 was only significant because of its imprinting effect and the QTL on SSC9, SSC10 and SSC16 were significant because of their dominance. For five QTL, three breed alleles could be identified and the order was always P over M over W. For the QTL with only two alleles, the alleles of breeds P and W or of P and M breeds were the same, but not for M and W breeds. For carcass weight, 16 QTL were mapped of which 13 showed a significant additive, seven a significant dominant and five a significant imprinting effect. For nine QTL, three different breed alleles could be identified and the order was always P over M over W.

Imprinting seemed to be important for these traits. When imprinting was not accounted for in the joint design, only eight, nine and nine QTL were mapped for respectively back fat depth, daily gain and carcass weight (not shown). Notably, all QTL found with the model without imprinting were also found when imprinting was considered (not shown). Imprinting was not always found in all breeds. For examples see Table 6, where estimated additive QTL effects are shown for traits with a significant imprinting effect. For example, the paternal allele effect of the P breed at the QTL for carcass weight on SSC7 was higher compared to the maternal allele effect, which pointed to maternal imprinting. This, however, was not observed in the M breed at this QTL (Table 6). The QTL on SSC3 for daily gain showed opposite modes of imprinting in the M and P breeds. Also no clear mode of imprinting could be observed for the imprinted QTL on SSC2. For the remaining QTL with imprinting effects the mode of imprinting was consistent (Table 6).

**Figure 1:**
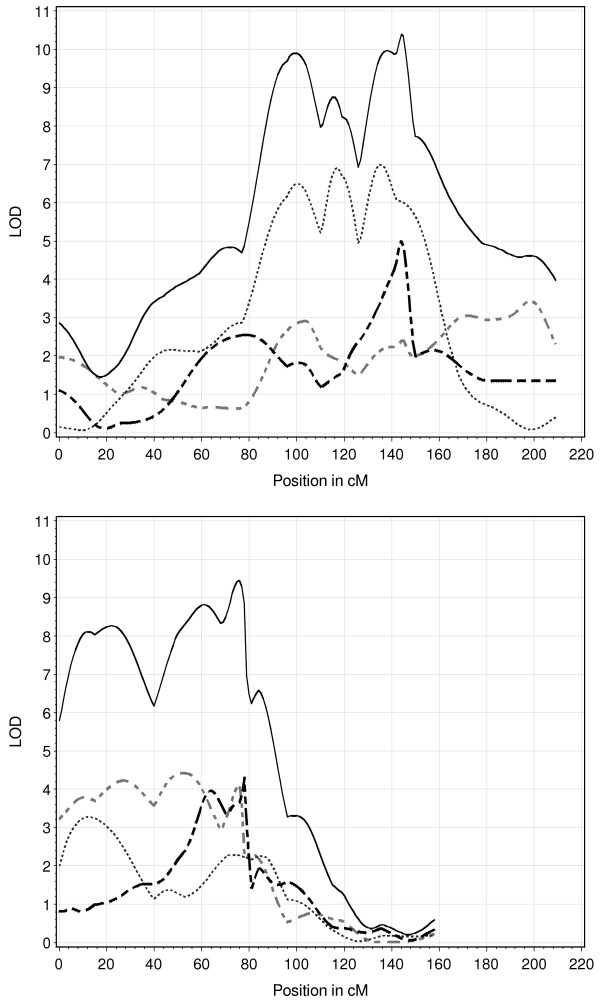
**LOD-score profiles for back fat depth on chromosome 1 (top) and on chromosome 2 (bottom)**. The solid black line denotes the results from the joint analysis; the dashed gray (small dotted, black dashed) line denotes the results of the MxP (WxP, WxM) analysis; the genetic map is given in the additional files.

When comparing the results of the joint design with those from the separate analysis of the crosses (Table 5) it can be observed that the number of significant QTL is much lower in the separate analysis, even if all QTL across the three crosses are considered as separate QTL. Additionally, in the joint design it was sometimes possible to map several QTL for one trait on one chromosome. For example, on SSC1 three QTL were detected for back fat depth in the joint design, whereas only one was detected within the single crosses. A comparison of the plots of the corresponding test statistics is given in Figure 1. The plot of the joint design is much sharper and more pronounced, leading to the separation of the three QTL. This can also be found on SSC2 for the same trait (Figure 1). On the one hand, in this case two QTL were found in the joint design, but one QTL in the designs MxP and WxM (Tables 2, 3, 4, and 5). On the other hand, almost all QTL detected in the single designs were also found in the joint design. This can be seen when comparing the overlap of the confidence intervals of the QTL (Tables 2, 3, 4, and 5).

When selecting QTL as cofactors, every QTL remained above its significance threshold level, and thus stayed in the model. For most QTL, the test statistic increased when additional QTL were selected as cofactors.

## Discussion

### QTL results

Because numerous QTL were mapped in the joint design, we will not discuss all identified QTL in detail. For a comparison of QTL found in this study and found by other groups see entries in the database pigQTLdb (Hu et al. [1]). Some QTL have also been reported by various other groups (e.g. QTL for carcass weight on SSC4). Other QTL are novel (e.g. QTL for back fat on SSC11 and SSC18). The signs of the breed effects are often, but not always, consistent with the history of the breed. For example, the Meishan breed is known to be a fatty breed, and it would subsequently be expected that most of the M breed allele effects at the QTL for back fat depth are higher compared to the P and W breed alleles. However, this was not always observed (Table 2). For daily gain and carcass weight traits, the breed allele effects of breed P are generally the highest (Tables 3 and 4), which fits to the breeding history of P. The P breed is frequently used as a sire line for meat production and daily gain and carcass weight are part of the breeding goal. Naturally, wild pigs have not been subject to artificial selection for the three traits; their breed allele effects were almost always lowest for the three traits (Tables 2, 3, and 4). Because the P breed was selected for increase in daily gain and carcass length and M is a much heavier and fattier breed than W, this was expected for daily gain and carcass length. Additionally, because P was selected against back fat during the last decades and W is a lean breed, the breed effects of M and P are frequently the same and lower than the fatty M breed allele effect (Table 2).

Three QTL with imprinting effects were found on SSC7 of which two were paternally imprinted. The mode of imprinting was not clear for imprinted carcass weight QTL (Table 6), because nearly the same paternal and maternal additive effects were observed in the M breed. De Koning et al. [20] have mapped a maternal expressed QTL for muscle depth on the same chromosome. A well known gene causing an imprinting effect is *IGF2*, which is located in the proximal region of SSC2 (Nezer et al. [21], van Laere et al. [22]). De Koning et al. [20] have mapped an imprinted QTL for back fat thickness with paternal expression close to the *IGF2 *region. In our study, we found an imprinted QTL in the corresponding chromosomal region for this trait as well (Tables 2 and 6), but it was not possible to unravel the mode of imprinting. A critical question is: are the detected imprinting effects really due to imprinting? As mentioned by Sandor and Georges [23] the number of imprinted genes in mammals has been estimated to be only around 100, which is not in a good agreement with the number of mapped imprinting QTL. The assumption underlying the classical model (4) for the detection of imprinting is that the F_1_-individuals are all heterozygous at the QTL. It has been shown by de Koning et al. [24] that in cases where this assumption is violated, the gene frequencies in the F_1_-sires and F_1_-dams may vary randomly, which might result in a significant, but erroneous, imprinting effect. This is especially a problem, when the number of males in the F_1_-generation is low, as in this study. The assumptions of model (4) and the pitfalls regarding imprinting effects do also hold in model (3). The additive effects were estimated depending on their parental origin, and if the F_1_-sires are not heterozygous at the QTL the estimates of the additive effects might differ depending on their parental origin, resulting in a significant imprinting effect. Hence, some cautions have to be made when drawing specific conclusions regarding the imprinting effects, especially for the imprinted QTL with an inconsistent mode of imprinting (Table 6). In some cases, imprinting effects might be spurious and due to within-founder breed segregation of QTL. Besides, the importance of imprinting for these traits has also been reported on a polygenic level within purebred pigs by Neugebauer et al. [25]. In addition, the same mode of imprinting in different founder alleles (Table 6) can be seen as evidence for real imprinting effects for these QTL.

### Experimental design and methods

When QTL experiments are analysed jointly, several requirements have to be fulfilled. Ideally, identical or to a large extent identical markers have to be genotyped in the designs and the allele coding has to be standardised. Subsequently, a common genetic map has to be established. Trait definition and measurement have to be standardised and, ideally, housing and rearing conditions of the animals should be the same or similar. All these points were fulfilled in the present study, since to a large extent the same markers were used, all animals were housed and slaughtered at one central unit and phenotypes were recorded by the same technical staff. Furthermore, due to the connectedness of the three designs, the situation for a combined analysis is especially favourable and allowed the use of model (3). Compared to a separate analysis, fewer parameters are estimated (i.e. seven instead of nine). Additionally the number of meioses used simultaneously was roughly three times higher. This led to the high statistical power of the joint design, which is confirmed by the large number of mapped QTL and by the reduced width of the confidence intervals. The high experimental power is probably due to the fact that not only the same founder breeds were used, but also to some extent the same founder animals within breeds. Hence the same founder alleles could be observed in the individuals of two F_2_-crosses, which increased the number of observations to estimate the effects. This is especially the case for the WxM and WxP crosses, which both go back to one and same W boar.

**Figure 2:**
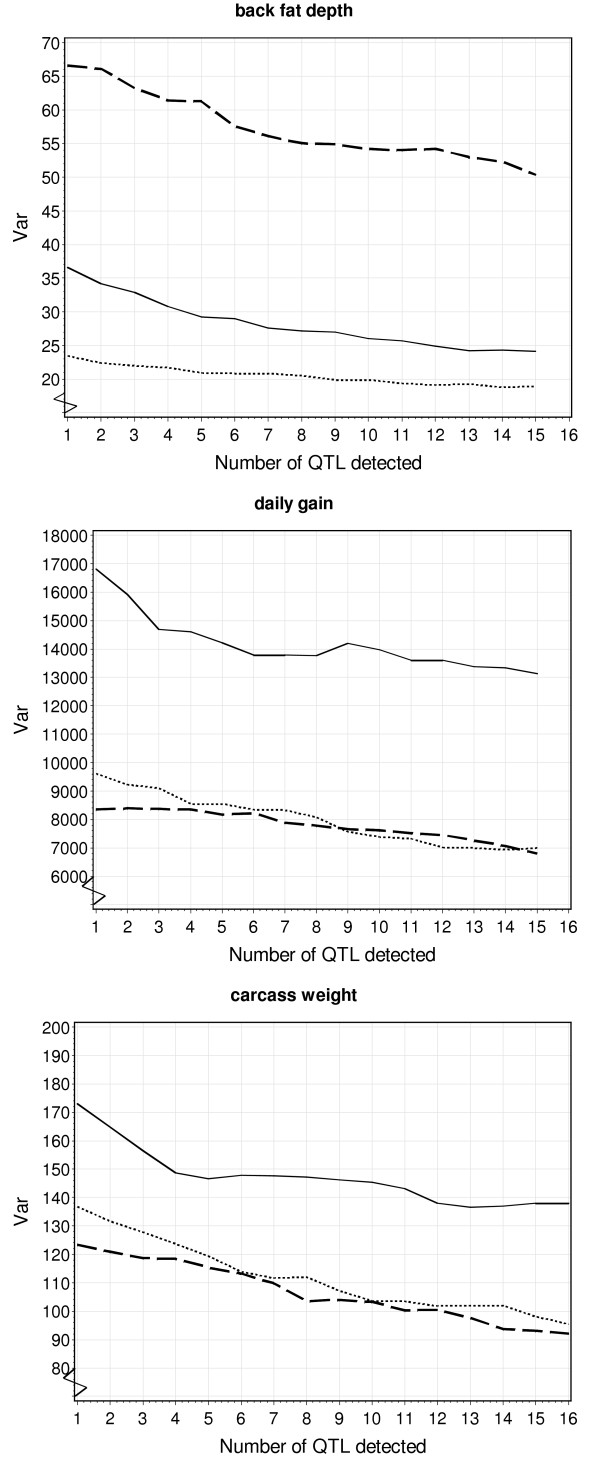
**Residual variance plotted against the number of QTL included in the model**. Solid line (dotted line, dashed line) denotes the MxP cross (WxP cross, WxM cross).

Model (3) was adapted from Liu and Zeng [10] but was extended for imprinting effects. Modelling imprinting seemed to be important for these traits. Ignoring imprinting resulted in a reduced number of mapped QTL for all three traits. Besides, all purely mendelian QTL (i.e. non-significant imprinting) were also found when imprinting was modelled. Hence, estimating two additional parameters in order to model imprinting obviously did not reduce the power to map purely mendelian QTL, favouring the model with imprinting. Thereby it was important to account for heterogeneous residual variances. A substantial heterogeneity was expected given the variation of the phenotypes within and across the three crosses (Table 1) and could be due to the different number of QTL segregating in the three crosses. Following this, it could be assumed that the heterogeneity would be reduced if more QTL were added as cofactors in the model. In Figure 2, the plots of the residual variances are shown for the three crosses and different number of QTL included in the model. It can be seen that the residual variances decreased and the differences became smaller, but did not disappear. One reason for this could be that there are still many more QTL segregating, which were not detected because their effects are too small. Indeed, Bennewitz and Meuwissen [26] have used QTL results from a separate analysis of the same three crosses to derive the distribution of QTL effects. They have shown that the additive QTL effects are exponentially distributed with many QTL of small effects. Model (3) was also flexible with regard to the number of QTL alleles, which was important given the large number of QTL with three different breed allele effects (Tables 2, 3, and 4).

Figure 2 also shows the benefit of including multiple QTL as cofactors in the model. The residual variances reduced continuously, which led to the increased statistical power and subsequently contributed to mapping the large number of QTL. The inclusion of QTL as cofactors is also known as composite interval mapping (CIM) and goes back to Zeng [27,28] and Jansen and Stam [29]. There are basically two main reasons for applying CIM. The first is to decrease residual variance and increase statistical power, as also used in this study. The second is to unravel a chromosomal position harbouring a QTL more precisely, i.e. to separate multiple closely linked QTL. This also requires scanning the chromosomal region of QTL identified in previous rounds of cofactor selection (in our study also rescanning confidence intervals of identified QTL), which, however, requires dense markers in those regions. Because marker density was not very high in this study, no attempts were made to detect multiple QTL within a QTL confidence interval. Low marker density should also be kept in mind when interpreting multiple QTL on single chromosomes, because the amount of information to separate them is limited.

The high statistical power is also due to the defined relative low significance level (i.e. 5% chromosome-wise). Hence, correction for multiple testing was done only for chromosomes or chromosome-segments and not for the whole genome or even for the whole experiment considering all three traits. The low significance level was chosen because a large number of QTL with small effects are segregating in this design [26], and many QTL with small effects would not have been found using a more stringent significance level. The downside of this strategy is, of course, that some mapped QTL will be false positives. The applied methods were computationally fast, mainly because of the applied regression approach, but also because the quick method was used [15] for the significance threshold determination rather than applying the permutation test. Piepho [15] has shown that this method is a good approximation if the data are normally distributed, which was the case in this study (not shown). Alternatively, a permutation test could have been used, which would result in more accurate threshold values and, as proposed by Rowe et al. [30,31], also for a more sophisticated identification of dominance and imprinting effects. This should be considered in putative follow-up studies.

## Conclusions

The present study showed the strength of analysing three connected F_2_-crosses jointly to map numerous QTL. The high statistical power of the experiment was due to the reduced number of estimated parameters and to the large number of individuals. The applied model was flexible with regard to the number of QTL and QTL alleles, mode of QTL inheritance, and was computationally fast. It will be applied to other traits and needs to be expanded to account for epistasis.

## Appendix

As stated in the main text, the restriction shown in eq (1) resulted in a re-parameterisation of the genetic model presented in eq (2). The re-parameterised model is as follows.

$$\left[\begin{array}{c} g_{MM}^{pm}\\ g_{PP}^{pm}\\ g_{WW}^{pm}\\ g_{MP}^{pm}\\ g_{PM}^{pm}\\ g_{MW}^{pm}\\ g_{WM}^{pm}\\ g_{WP}^{pm}\\ g_{PW}^{pm} \end{array}\right]=\left[\begin{array}{ccccccc} 1 & 1 & 0 & 0 & 0 & 0 & 0\\ 0 & 0 & 1 & 1 & 0 & 0 & 0\\ -1 & -1 & -1 & -1 & 0 & 0 & 0\\ 1 & 0 & 0 & 1 & 1 & 0 & 0\\ 0 & 1 & 1 & 0 & 1 & 0 & 0\\ 1 & -1 & 0 & -1 & 0 & 1 & 0\\ -1 & 1 & -1 & 0 & 0 & 1 & 0\\ -1 & 0 & -1 & 1 & 0 & 0 & 1\\ 0 & -1 & 1 & -1 & 0 & 0 & 1 \end{array}\right]\left[\begin{array}{c} a_{i}^{p}\\ a_{i}^{m}\\ a_{j}^{p}\\ a_{j}^{m}\\ d_{MP}\\ d_{MW}\\ d_{PW} \end{array}\right]+\left[\begin{array}{c} \mu \\ \mu \\ \mu \\ \mu \\ \mu \\ \mu \\ \mu \\ \mu \\ \mu \end{array}\right]$$

The upper subscripts denote or the parental origin (i.e. either paternal (*p*) or maternal (*m*)) and the lower subscripts denote the breed origin M, P, and W. This model contained only four additive effects (two paternal and two maternal). Using the above notation, ${\hat{a}}_{M}^{p}={\hat{a}}_{i}^{p}$, ${\hat{a}}_{P}^{p}={\hat{a}}_{j}^{p}$ and ${\hat{a}}_{W}^{p}=-({\hat{a}}_{i}^{p}+{\hat{a}}_{j}^{p})$. The same holds for the maternal alleles. The applied regression model (eq (3) in the main text) estimated the four additive effects for the breeds M and P. The two effects for W not modelled were reconstructed, as shown above.

## Competing interests

The authors declare that they have no competing interests.

## Authors' contributions

CR did the statistical analysis and JB developed the models. Both authors drafted the manuscript. Both authors read and approved the final manuscript.

## Supplementary Material

Additional file 1 **Genetic map (marker name and distance from the start of the chromosome)**. The genetic map, including the marker names and the distance from the start of the chromosome. Click here for file
