# Supplementary material for: Joint QTL analysis of three connected F2-crosses in pigs
Source: Genet Sel Evol. 2010 Nov 1;42(1):40. doi: 10.1186/1297-9686-42-40 (PMC2988712; doi:10.1186/1297-9686-42-40)
Supplement: Additional file 1 — Genetic map (marker name and distance from the start of the chromosome). The genetic map, including the marker names and the distance from the start of the chromosome. [file 1297-9686-42-40-S1.PDF]

SSC1: SW1514 [0.0]; SWR485 [25.4], SWR2300 [43.5]; S0008 [59.3]; SW2130 [77.3]; EEF1A1 [95.8]; IGFR [104.1]; SW307 [110.3]; S0082 [119.2]; SW780 [126.3]; SW803 [141.7]; TPM2 [144.7]; TGFBR1 [149.6]; SW705 [178.5]; EAA [209.1]

SSC2: SW2443 [0.0]; SWC9 [5.2]; SW2623 [14.9]; S0141 [39.9]; SW240 [52.9]; MLP [68.0]; MYOD1 [70.6]; MEF2B [76.5]; UBL5 [77.8]; RETN [78.3]; INSR [78.3]; SW395 [81.0]; CDF [84.3]; S0010 [96.0]; S0378 [115.1]; FBN2 [119.4]; SW2192 [135.5]; S0036 [158.4]

SSC3: SERPINE1 [0.0]; SW72 [11.6]; S0206 [35.9]; ASPN [50.2]; OIF [50.8]; SW902 [57.9]; SW828 [74.0]; SW314 [104.6]; LPW [116.1]; SW [138.6]

SSC4: SW489 [0.0]; CMYC [19.7]; SW835 [27.7]; SWR73 [43.6]; SW2128 [50.9]; S0145 [50.9]; SW1073 [62.1]; SW1089 [67.3]; VATP [69.1]; ATP1B1 [71.6]; S0073 [75.3]; ATF6 [78.5]; OCT1 [79.1]; HSD17B7 [79.9]; SDHC [80.1]; MPZ [80.1]; APOA2 [80.2]; CASQ1 [81.0]; ATP1A2 [81.8]; MEF2D [82.5]; LMNA [84.6]; GBA [85.1]; PKLR [85.6]; IVL [87.6]; EAL [93.7]; ATP1A1 [95.8]; TSHB [98.1]; NGFB [99.6]; AMPD1 [100.2]; SW2435 [107.7]; AGL [121.5]; S0097 [135.9]; PXMP1 [142.8]; CNN3 [142.8]

SSC5: SW413 [0.0]; SWR453 [39.0]; SW2425 [53.0]; SW2 [64.4]; S0005 [77.3]; SW152 [92.2]; IGF1 [110.0]; SW995 [120.1]; DCN [131.5]; MYF5_DDEI [150.4]; SW967 [157.9]

SSC6: S0035 [0.0]; SW1329 [24.8]; SW1057 [58.1]; FTO [73.7]; S0087 [80.0]; ETH5001 [94.4]; RYR [96.4]; LIPE [98.3]; TGFB1 [99.5]; A1BG [101.2]; EAH [102.4]; SKI [106.0]; BNP1 [112.0]; HFABP [124.9]; ID3 [127.1]; S0146 [141.5]; S0003 [150.4]; SW824 [165.7]; LERP [177.9]; P3 [207.8]; EAO [235.5]

SSC7: S0025 [0.0]; S0064 [36.3]; SWR1078 [50.0]; ID4_ECO [61.3]; ID4_SMA [61.3]; CYPD [73.3]; CYPA [73.3]; KE6 [75.2]; TNFA [75.5]; TNFB [76.2]; S0102 [86.5]; PSMA4 [100.9]; PLIN [106.8]; S0066 [113.0]; S0115 [143.3]; FOS [149.7]; SW581 [173.9]; S0212 [196.7]; AACT2 [206.0]; PO1A [206.2]; PI2 [208.8]; IGH2 [229.5]

SSC8: SW905 [0.0]; PGCMUT [18.0]; SW933 [34.0]; SW1070 [49.4]; S0144 [85.0]; SW16 [110.1]; SW61 [127.1]; OPN [151.8]

SSC9: EAK [0.0]; HPX [19.8]; SW21 [28.7]; SW911 [59.1]; SLN [71.0]; SW2074 [80.0]; APOA1 [89.5]; LPR [110.1]; EAN [113.0]; PDK4 [113.8]; PDK4i [113.8]; PDK41 [113.8]; IL6 [117.1]; VISF [125.6]; VISF_PRO [125.6]; PRKAR2B [127.3]; PIC3CG [127.3]; MYOG [130.9]; SW1435 [132.5]; SW2093 [135.6]; GLUL [147.5]; SW174 [158.1]; S0114 [161.3]; EAE [187.4]; SW1349 [194.6]

SSC10: SW830 [0.0]; SW443 [30.6]; SW497 [52.5]; GAS1 [74.1]; SWR1849 [82.7]; SW2000 [105.7]; SW1708 [125.0]; SW2067 [150.8]

SSC11: S0392 [0.0]; POSTN [22.6]; SW1632 [28.4]; SW435 [61.0]; SW1827 [93.3]

SSC12: S0143 [0.0]; EAD [10.8]; SW957 [32.0]; GH1-H [40.7]; GH1-A ; S0083 [51.0]; SW874 [64.5]; S0090 [84.1]; S0147 [99.3]; S0106 [109.8]; SWR1021 [127.1]; SW605 [137.9]

SSC13: S0282 [0.0]; S0076 [39.2]; SW864 [60.8]; SWR1008 [70.7]; TF [81.2]; S0068 [94.2]; POU1F1 [108.2]; SW520 [120.5]; SW38 [152.6]; S0215 [179.0]; CSTB [204.4]

SSC14: EDG3 [0.0]; SW857 [27.5]; SW2038 [43.8]; SW540 [60.7]; ACTN2 [70.6]; ACTA1 [78.0]; SW210 [84.3]; SW2488 [105.1]; SW55 [122.1]; SW2515 [151.2]

SSC15: KS169 [0.0]; S0148 [21.2]; EAG [31.3]; SW964 [41.9]; SW15 [52.5]; SW2053 [71.9]; SW1983 [99.4]

SSC16: S0111 [0.0]; SW1035 [21.2]; SW419 [33.3]; S0077 [43.9]; S0026 [61.5]; SWR2480 [69.4]; SPARC [78.4]; S0061 [98.0]

SSC17: SW335 [0.0]; SW1891 [6.5]; S0296 [15.6]; SW1920 [41.3]; GHRH [43.6]; RNPC2 [45.4]; SJ063 [69.9]; GNAS [86.4]; EEF1A2 [94.6]; SW2427 [97.9]

SSC18: SW1808 [0.0]; EAI [10.9]; LEPTIN [33.5]; SW787 [43.6]; S0062 [58.8]; GCK [71.2]
